# Supramolecular hydrogels formed from poly(viologen) cross-linked with cyclodextrin dimers and their physical properties

**DOI:** 10.3762/bjoc.8.182

**Published:** 2012-09-20

**Authors:** Yoshinori Takashima, Yang Yuting, Miyuki Otsubo, Hiroyasu Yamaguchi, Akira Harada

**Affiliations:** 1 Department of Macromolecular Science, Graduate School of Science, Osaka University, Toyonaka, Osaka 560-0043, Japan

**Keywords:** cyclodextrins, poly(viologen), supramolecular hydrogel

## Abstract

Supramolecular materials with noncovalent bonds have attracted much attention due to their exclusive properties differentiating them from materials formed solely by covalent bonds. Especially interesting are rotor molecules of topological complexes that shuttle along a polymer chain. The shuttling of these molecules should greatly improve the tension strength. Our research employs cyclodextrin (CD) as a host molecule, because CD effectively forms polyrotaxanes with polymers. Herein we report the formation of supramolecular hydrogels with an α-CD dimer (α,α-CD dimer) as a topological linker molecule, and a viologen polymer (VP) as the polymer chain. The supramolecular hydrogel of α,α-CD dimer/VP forms a self-standing gel, which does not relax (*G'* > *G''*) in the frequency range 0.01–10 rad·s^−1^. On the other hand, the supramolecular hydrogel decomposes upon addition of bispyridyl decamethylene (PyC_10_Py) as a competitive guest. Moreover, the β-CD dimer (β,β-CD dimer) with VP does not form a supramolecular hydrogel, indicating that complexation between the C_10_ unit of VP and the α-CD unit of the α,α-CD dimer plays an important role in the formation of supramolecular hydrogels.

## Introduction

Development of functional soft materials has attracted much attention due to the numerous practical applications [1–3]. Typically, soft materials fall into one of two types of gels: physical gels and chemical gels [4–9]. Recently, topological cross-linked polyrotaxanes have been identified as tertiary gels, which should create a new paradigm in materials science [10]. Polyrotaxanes form topological gels, because the rotor molecules, which act as cross-linkers, slide on the axial polymer chain. In contrast, chemical gels do not exhibit cross-linker slippage.

Previously, there have been some reports of supramolecular complexes with cyclodextrin (CD) dimers. A supramolecular hydrogel, which was constructed by the formation of an inclusion complex between the copolymer with an adamantyl group and CD dimer, showed a lower critical solution temperature (LCST) [11]. Another report indicated that adding selenium or platinum complexes yields supramolecular assemblies of bis(molecular tube)s cross-linked with the β-CD dimer, which form nanofibers [12–15]. Moreover, mechanically linked polyrotaxane with the α-CD and poly(ethylene glycol) (PEG) produces a hydrogel material, which exhibits unique physical properties [10].

Previously, we have prepared a polyrotaxane using α-CD and PEG [16–17]. The α-CD/PEG polyrotaxane forms a hydrogel material in high concentrations [18]. Using polyelectrolytes as threading molecules results in complexation between the polyelectrolyte and α-CD within the range of the ^1^H NMR time scale due to the slow equilibrium [19–20]. Cationic groups, such as pyridinium and pyridylpyridinium terminal groups, inhibit the decomposition of polyrotaxane and stabilize the complexes between α-CD and cationic alkanediyl compounds [21–23]. Herein, to study the formation of supramolecular hydrogels with the α,α-CD dimer, we chose the viologen polymer (VP), which possesses multiple cations, as the axis molecules. Decamethylene units function as recognition sites of α-CD, and bipyridyls work as electric barriers.

## Results and Discussion

### Preparation of CD dimers and viologen derivatives

**Figure 1:**
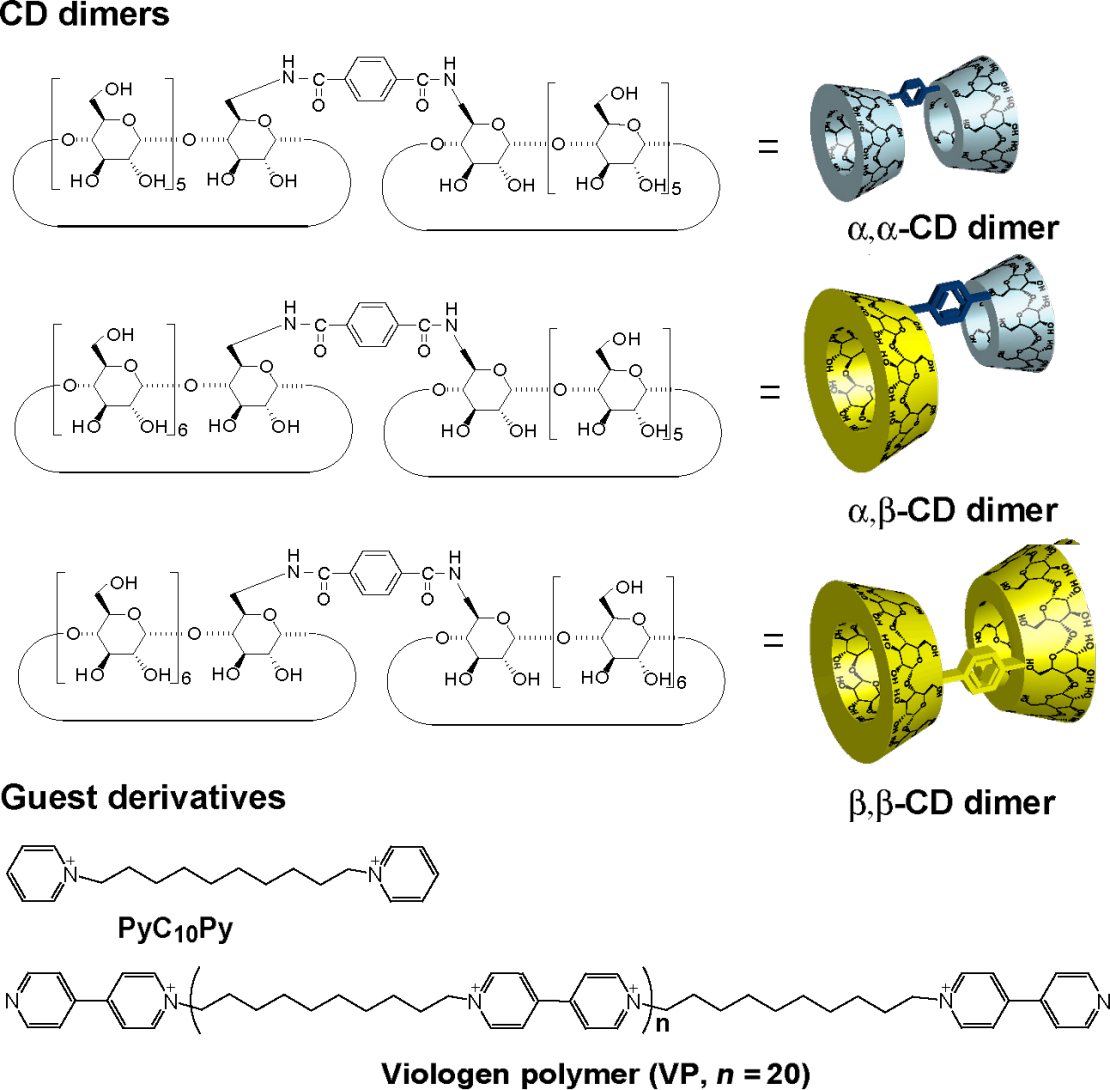
Chemical structures of the CD dimer (α,α-CD dimer, α,β-CD dimer, and β,β-CD dimer) and guest derivatives [PyC_10_Py and viologen polymer (VP)]. Bromonium anions are omitted in guest derivatives.

Figure 1 depicts the chemical structures of the cyclodextrin dimers (α,α-CD dimer, α,β-CD dimer, and β,β-CD dimer) and pyridyl derivatives (PyC_10_Py and viologen polymer (VP)). The α,α-CD and β,β-CD dimers are prepared by reacting the corresponding 6-amino-CDs and terephthalic acid using 4-(4,6-dimethoxy-1,3,5-triazin-2-yl)-4-methylmorpholinium chloride *n*-hydrate (DMT-MM) as a condensing reagent in DMF. The α,β-CD dimer is prepared by reacting 6-amino-α-CD and 6-*O*-(4-carboxylphenylamide)-β-CD using DMT-MM in DMF. These CD dimers are purified by preparative reversed-phase chromatography using DIAION HP-20 beads. As described in the experimental section, the reaction of 1,10-dibromodecane with 4,4’-bipyridyl in DMF gives VP, where the number of VP units is 20 and was determined by the ratio of integral values of the end group and the main chain unit in the ^1^H NMR spectrum.

### Hydrogelation between the CD dimer and viologen polymer

**Figure 2:**
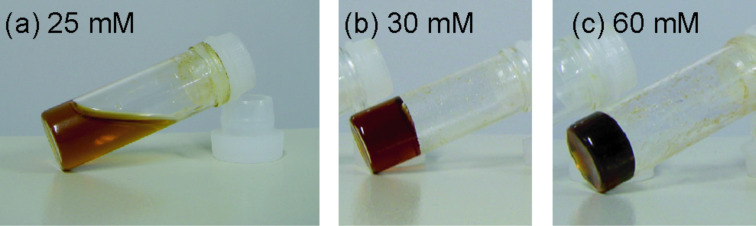
Photographs of hydrogelation with various concentrations of α,α-CD dimer/VP in water. Aqueous solution of α,α-CD dimer/VP forms the hydrogel at concentrations above 30 mM.

Mixing the α,α-CD dimer and VP in aqueous solutions at room temperature slightly increases the viscosity of the α,α-CD dimer/VP, but hydrogels are not formed. On the other hand, after heating at 100 °C for 7 h, an aqueous solution of the α,α-CD dimer/VP forms a supramolecular hydrogel containing over 30 mM (VP unit/CD unit 4:1) (Figure 2). The bipyridyl group of VP functions as an electric barrier, which prevents threading and dethreading of the α-CD unit in the α,α-CD dimer onto the decamethylene unit of VP at 30 °C; this observation suggests that the α-CD unit of the α,α-CD dimer cannot exceed the electric barrier at 30 °C, whereas after heating at 100 °C, the α-CD unit exceeds the barrier to form polyrotaxanes.

**Figure 3:**
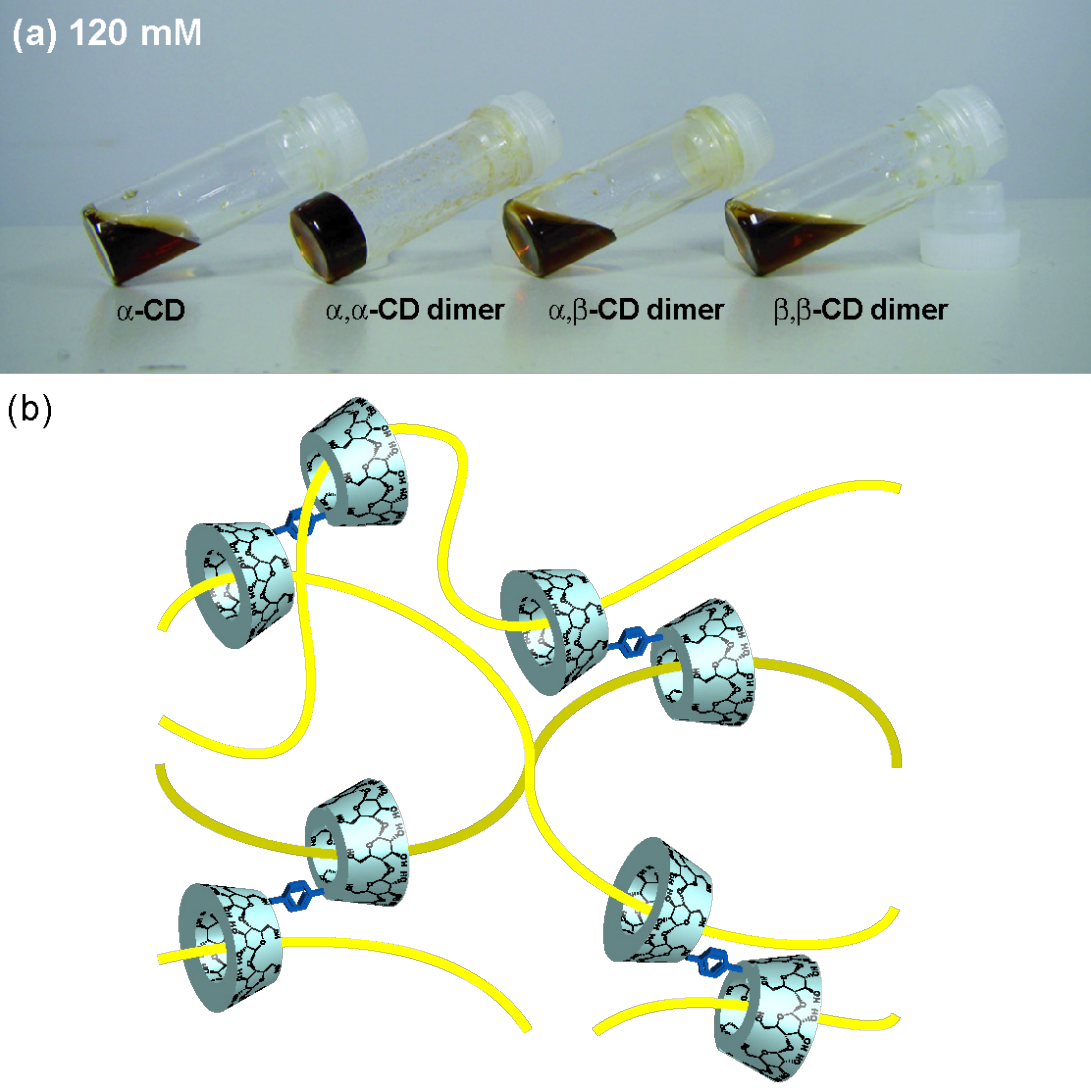
Hydrogelation of VP with various CD derivatives (VP unit/CD 4:1) at 25 °C. (a) Concentrations of CDs are 120 mM. (b) Proposed structure of the α,α-CD dimer/VP supramolecular hydrogel.

To confirm complementarity between the CD dimer and VP, we then investigated the formation of supramolecular hydrogels of VP with the α,β-CD dimer and the β,β-CD dimer. For each sample, the CD concentration was adjusted to 120 mM (VP unit/CD unit 4:1). Even with a dimer/VP concentration greater than 60 mM, the α,β-CD dimer/VP and β,β-CD dimer/VP do not form supramolecular hydrogels. These results indicate that complexation between the C_10_ unit of VP and the α-CD unit of the α,α-CD dimer is important for the formation of cross-links between VPs (Figure 3). The cavity size of β-CD is too large to allow formation of a stable cross-linked polyrotaxane complex. The α,β-CD dimer and β,β-CD dimer do not function as crosslinking molecules between VP and β-CD. Actually, the association constant of α-CD with decamethylene is much higher than that of β-CD with decamethylene [24]. Consequently, the association constant plays an important role in gel formation.

To confirm a supramolecular hydrogel formed by crosslinking VP with the α,α-CD dimer, we added PyC_10_Py as a competitive guest to the supramolecular hydrogel of the α,α-CD dimer/VP. After adding PyC_10_Py (PyC_10_Py/VP unit/α,α-CD dimer 8:8:1) and heating at 100 °C, the supramolecular hydrogel of the α,α-CD dimer/VP changes to the sol even at a high concentration ([α,α-CD dimer] = 60 mM), because the α-CD unit of the α,α-CD dimer forms an inclusion complex with PyC_10_Py. This inclusion-complex formation causes the cross-links between the α,α-CD dimer and VP to decompose.

### ^1^H NMR study of complexation of the α,α-CD dimer/VP

**Figure 4:**
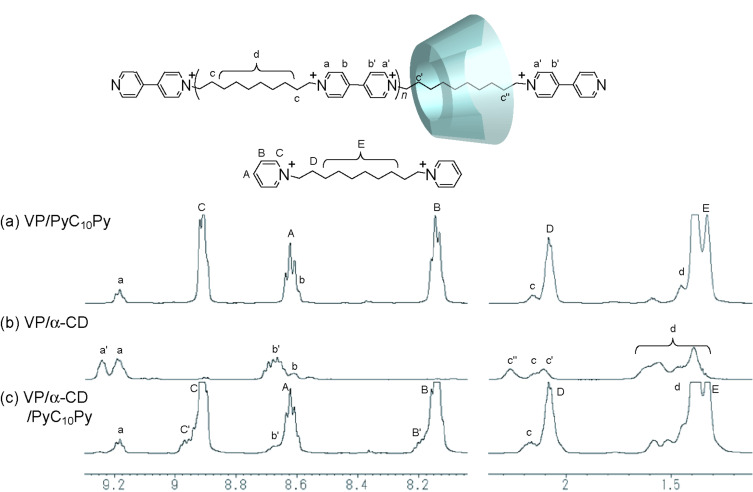
500 MHz ^1^H NMR spectra of VP (VP unit 2 mM) with α-CD and PyC_10_Py (VP unit/CD/PyC_10_Py 1:2:8) in D_2_O at 30 ^o^C: (a) VP and PyC_10_Py, (b) VP and α-CD, and (c) VP, PyC_10_Py, and α-CD.

To observe the competitive effect of PyC_10_Py, we conducted ^1^H NMR studies on complexation between α-CD/VP and a competitive experiment using PyC_10_Py. Figure 4 shows ^1^H NMR spectra of VP/PyC_10_Py, VP/α-CD, and VP/α-CD/PyC_10_Py. Addition of α-CD causes peak splitting of the decamethylene and pyridyl protons of VP (VP unit/α-CD 1:2), indicating the formation of a polyrotaxane VP/α-CD complex. The association–dissociation equilibrium between VP and α-CD is slow on the NMR time scale. On the other hand, upon addition of PyC_10_Py to the VP/α-CD complex (VP unit/α-CD/PyC_10_Py 1:2:8), the splitting peaks of the VP/α-CD complex disappear, and then signals of PyC_10_Py split due to complexation between PyC_10_Py and α-CD (Figure 4c). These results indicate that the excess PyC_10_Py disturbs complexation of α-CD and VP. The sol state of the α,α-CD dimer/VP in the presence of PyC_10_Py is attributed to the dissociation of VP and the α,α-CD dimer, suggesting that complexation between VP and the α-CD units is necessary to form the gel.

### Viscoelastic property of the α,α-CD dimer/VP hydrogel

**Figure 5:**
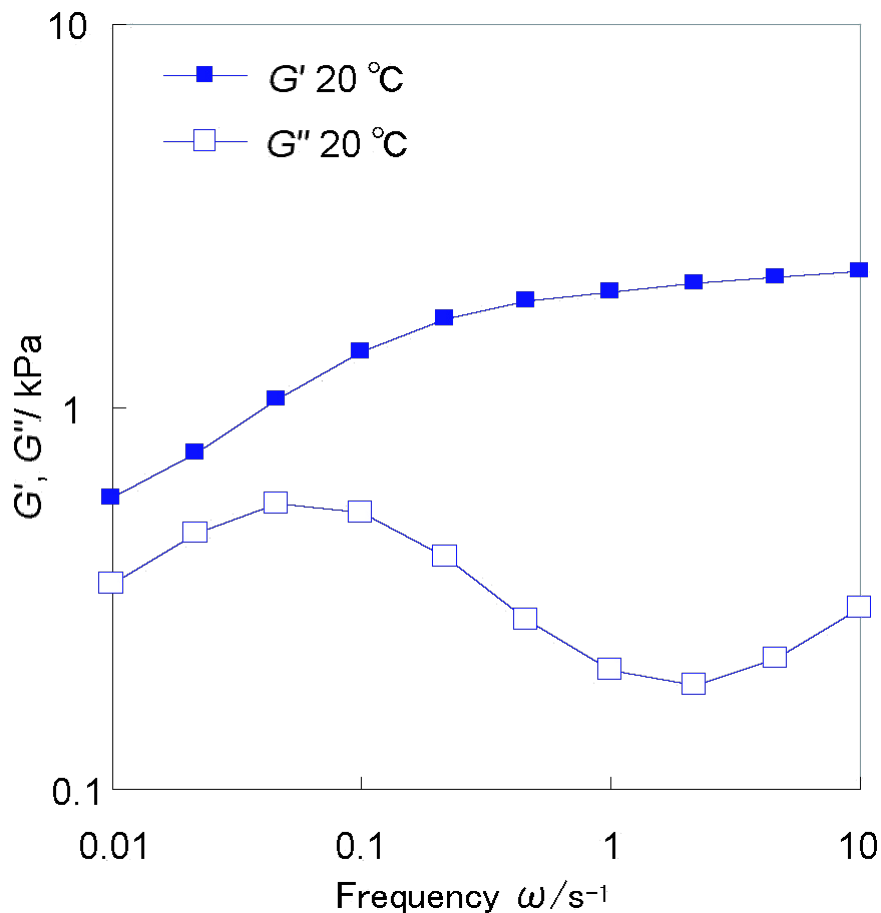
*G'* and *G''* of the α,α-CD dimer/VP hydrogel as a function of frequency (ω). Applied shear strain amplitude is 1%.

Figure 5 shows the storage elastic modulus (*G'*) and loss elastic modulus (*G''*) for an α,α-CD dimer/VP hydrogel (60 mM) at 20 °C. The master curve of the hydrogel is similar to the Voigt Model. *G''* relaxes as the frequency increases. However, the hydrogel does not relax (*G'* > *G''*) in the frequency range 0.01–10 rad·s^−1^, indicating a self-standing gel. This behavior are similar to chemically cross-linked gels even though the α,α-CD dimer/VP hydrogel is topologically cross-linked between VPs with the α,α-CD dimer. This result confirms that complexation of VP and the α,α-CD dimer is stable and responsible for the stability of the hydrogel.

## Conclusion

Mixing VP and the α,α-CD dimer creates a hydrogel, which is expected to realize supramolecular materials with a high tensile strength and self-healing abilities. The complementarity between α-CD and the decamethylene units plays an important role in the formation of supramolecular hydrogels composed of α,α-CD dimer/VP. VP has an electric barrier between the decamethylene units, which is a unique feature of this supramolecular hydrogel. The electric barrier prevents dethreading of α-CD from VP, yielding a self-standing supramolecular hydrogel. We will electrochemically control the elasticity of the α,α-CD dimer/VP hydrogel.

## Experimental

### Preparation of α,α-CD dimer


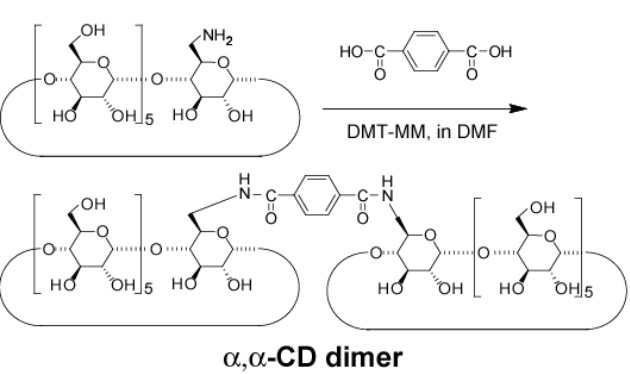


6-NH_2_-α-CD (120 mg, 0.12 mmol) and terephthalic acid (8 mg, 0.50 mmol) were dissolved in dried DMF (20 mL). DMT-MM (34 mg, 0.12 mmol) was added and the mixture was stirred at rt for 4 days. After evaporation of the solvent, the residue was dissolved in water (10 mL) and poured into acetone (100 mL). The product was collected and purified by reversed-phase chromatography (elution: water–acetonitrile) to give α,α-CD dimer as a white solid in 22% yield. ^1^H NMR (DMSO-*d_6_*, 500 MHz) δ 8.36 (t, 2H, -N*H*), 7.88 (s, 4H, *Ph*), 5.59–5.40 (m, 24H, O(2,3)*H* of α-CD), 4.97–4.78 (m, 12H, C(1)*H* of α-CD), 4.55–4.41 (m, 10H, O(6)*H* of α-CD), 3.84–3.48 (m, C(3,6,5,3,4)*H* of α-CD); MALDI–TOF *m*/*z*: 2095 [M + Na]^+^.

### Preparation of α,β-CD dimer

#### a) Methyl terephthalate-β-CD


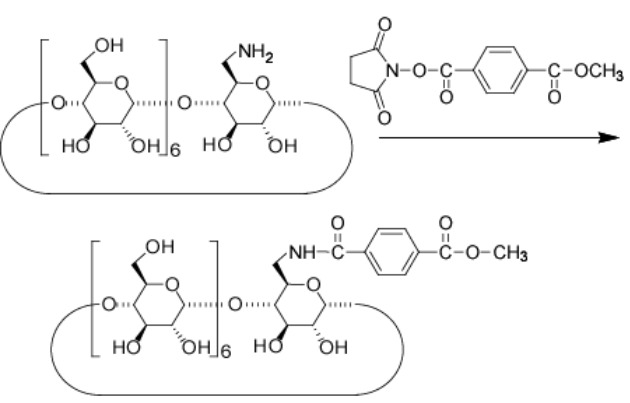


To a solution of 6-NH_2_-β-CD (566 mg, 0.50 mmol) in dried DMF (7 mL) was added methyl terephthalate succinimidyl ester (137.6 mg, 0.50 mmol). After stirring for 2 days at rt, the solution was poured into acetone (100 mL) to give methyl terephthalate-β-CD as a yellow solid in 43% yield. ^1^H NMR (DMSO-*d*_6_, 500 MHz) δ 8.46 (t, 1H, -N*H*), 8.00 (d, 2H, *Ph*), 7.95 (s, 3H, -C*H*_3_), 7.94 (d, 2H, *Ph*), 5.83–5.59 (m, 14H, O(2,3)*H* of β-CD), 4.95–4.79 (m, 7H, C(1)*H* of β-CD), 4.45–4.32 (m, 6H, O(6)*H* of β-CD), 3.74–3.51 (m, C(3,6,5,3,4)*H* of α-CD); TLC: *R*_f_ 0.22 (*n*-butanol/ethanol/water 5:4:3).

#### b) Terephthalic acid-β-CD


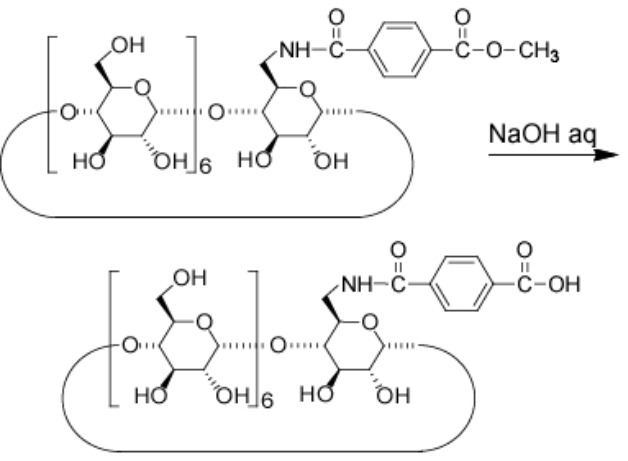


To a solution of methyl terephthalate-β-CD (605 mg, 0.47 mmol) in water (120 mL) was added NaOH (0.1 M, 7 mL). After stirring for 12 hours at rt, the solution was concentrated and purified by DIAION HP-20 column. The column was flushed with water (500 mL) and then eluted with water/methanol 80:20 (v/v). The fraction was concentrated to give terephthalic acid-β-CD as a yellow solid in 70% yield. ^1^H NMR (DMSO-*d*_6_, 500 MHz) δ 8.39 (t, 1H, -NH), 7.98 (d, 2H, Ph), 7.90 (d, 2H, Ph), 5.83–5.59 (m, 14H, O(2,3)*H* of β-CD), 4.95–4.79 (m, 7H, C(1)*H* of β-CD), 4.45–4.32 (m, 6H, O(6)*H* of β-CD), 3.74–3.51 (m, C(3,6,5,3,4)*H* of α-CD); TLC: *R*_f_ 0.32 (*n*-butanol/ethanol/water 5:4:3).

#### c) α,β-CD dimer


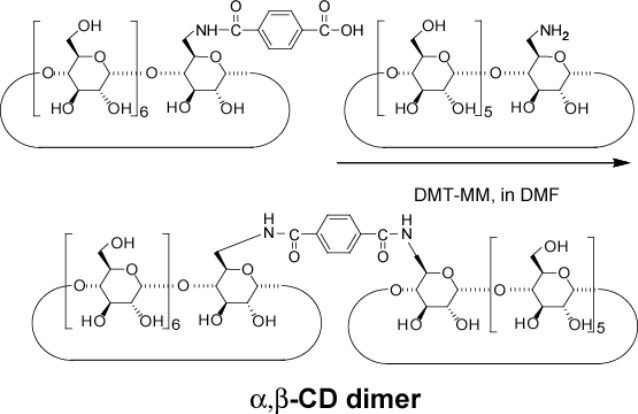


The synthetic procedure was the same as α,α-CD dimer, using terephthalic acid-β-CD (65 mg, 50 μmol), 6-NH_2_-α-CD (59 mg, 60 μmol), DMT-MM (17 mg, 60 μmol), dried DMF (8 mL) to give α,β-CD dimer in 36% yield as a white solid. ^1^H NMR (DMSO-*d*_6_, 500 MHz) δ 8.32, 8.27 (m, 2H, -NH), 7.89 (s, 4H, Ph), 5.80–5.44 (m, 26H, O(2,3)*H* of CDs), 4.97–4.78 (m, 13H, C(1)*H* of CDs), 4.53–4.35 (m, 11H, O(6)*H* of CDs), 3.86–3.37 (m, C(3,6,5,3,4)*H* of α-CD); TLC: *R*_f_ 0.04 (*n*-butanol/ethanol/water 5:4:3); MALDI–TOF *m*/*z*: 2259 [M + Na]^+^.

### Preparation of β,β-dimer

β,β-Dimer was prepared according to our previous report [25].

### Preparation of viologen polymer (VP)

1,10-Dibromodecane (7.3 g, 24 mmol) was added to a solution of 4,4’-bipyridyl (4 g, 24 mmol) in DMSO (40 mL). After being stirred at 100 °C for 2 d, the solution became turbid. The precipitate was collected and washed with acetone three times. The product was purified by dialysis for 4 d to give VP in 20% yield as a brown solid. ^1^H NMR (D_2_O, 500 MHz) δ 9.25 (m, 92H, 2-position of bipyridyl in the middle of the axle), 9.14 (m, 4H, 2-position of bipyridyl at the end of the axle near the decamethylene part), 8.94 (m, 4H, 2-position of bipyridyl at the end of the axle apart from the decamethylene part), 8.69 (m, 92H, 3-position of bipyridyl in the middle of the axle), 8.58 (m, 4H, 3-position of bipyridyl at the end of the axle near the decamethylene part), 8.18 (m, 4H, 3-position of bipyridyl at the end of the axle apart from the decamethylene part), 4.85 (m, 96H, α methylene in decamethylene), 2.21 (m, 96H, β methylene in decamethylene ), 1.72–1.30 (m, 288H, χ, δ, ε methylene in decamethylene).

### Preparation of [Py-(CH_2_)_10_-Py]^2+^·2Br^−^ (PyC_10_Py)

Pyridine (158 mg, 2.0 mmol) and 1,10-dibromodecane (315 mg, 0.80 mmol) were dissolved in acetone and heated under reflux for 3 d. After evaporation of the solvent, the residue was dissolved in methanol (20 mL) and poured into diethyl ether (200 mL). The product was collected by centrifugation to give PyC_10_Py in 91% yield as a brown solid. ^1^H NMR (D_2_O, 500 MHz) δ 8.90 (d, *J* = 6.6 Hz, 4H, 2-position of pyridine), 8.62 (t, *J* = 8.2 Hz, 2H, 4-positon of pyridine), 8.14 (t, *J* = 7.7 Hz, 4H, 3-positon of pyridine), 4.67 (t, *J* = 7.3 Hz, 4H, α methylene in decamethylene), 2.08 (m, 4H, β methylene in decamethylene), 1.42–1.30 (m, 12H, χ, δ, ε methylene in decamethylene).

### Rheological measurements

Dynamic viscoelasticity were measured by using an Anton Paar MCR301 rheometer at a strain of 0.1%. The storage elastic modulus (*G'*) and loss elastic modulus (*G''*) were measured at 20 °C. The sample concentration was adjusted to 1.0 wt %.

## Supporting Information

File 1 Additional information and ^1^H NMR spectra of all new compounds.
