# Supplementary material for: Supramolecular hydrogels formed from poly(viologen) cross-linked with cyclodextrin dimers and their physical properties
Source: Beilstein J Org Chem. 2012 Sep 20;8:1594–600. doi: 10.3762/bjoc.8.182 (PMC3510991; doi:10.3762/bjoc.8.182)
Supplement: File 1 — Additional information and 1H NMR spectra of all new compounds. [file Beilstein_J_Org_Chem-08-1594-s001.pdf]

Supporting Information

for

Supramolecular hydrogels formed from poly(viologen) cross-linked with cyclodextrin dimers and their physical properties

Yoshinori Takashima, Yang Yuting, Miyuki Otsubo, Hiroyasu Yamaguchi, Akira Harada*,§

Address: Department of Macromolecular Science, Graduate School of Science, Osaka University, Toyonaka, Osaka 560-0043, Japan

Email: Akira Harada - harada@chem.sci.osaka-u.ac.jp

* Corresponding author

§Telephone +81-6-6850-5445; Fax +81-6-6850-5445

Additional information and ^{1}H NMR spectra of all new compounds

Preparation of viologen polymer (VP)

1,10-Dibromodecane (7.3 g, 24 mmol) was added to a solution of 4,4'-bipyridyl (4.0 g, 24 mmol) in DMF (40 ml). After being stirred at 100 °C for 2 days, the solution became turbid. The precipitate was collected and washed with acetone three times. The product was purified by dialysis for 4 d to give VP in 20% yield as a brown solid.

^{1}H NMR (D_2O, 500 MHz): δ 9.25 (m, 2-position of bipyridyl in the middle of the axle), 9.14 (m, 4H, 2-position of bipyridyl at the end of the axle near the decamethylene part), 8.94 (m, 4H, 2-position of bipyridyl at the end of the axle apart from the decamethylene part), 8.69 (m, 3-position of bipyridyl in the middle of the axle), 8.58 (m, 4H, 3-position of bipyridyl at the end of the axle near the decamethylene part), 8.18 (m, 4H, 3-position of bipyridyl at the end of the axle apart from the decamethylene part), 4.85 (m, α methylene in decamethylene), 2.21 (m, β methylene in decamethylene), 1.72–1.30 (m, χ, δ, ε methylene in decamethylene).

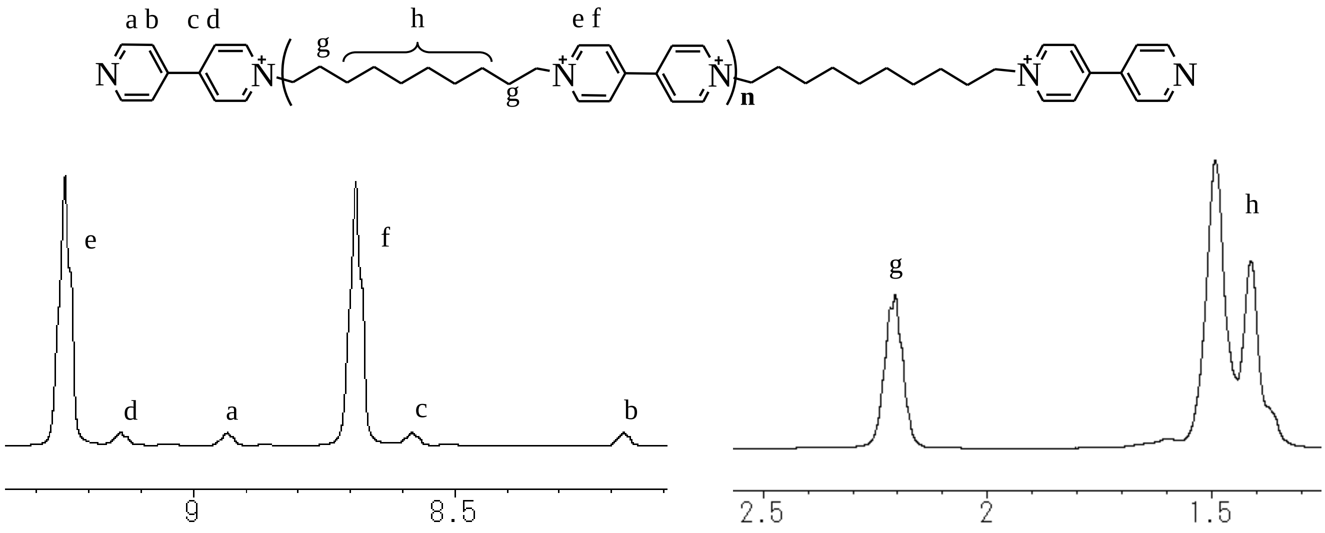

Figure S1: 500 MHz ^{1}H NMR spectra of VP in D_2O at 30 °C.

Preparation of $PyC_{10}Py$

Pyridine (158 mg, 2.0 mmol) and 1,10-dibromodecane (315 mg, 0.80 mmol) were dissolved in acetone and refluxed for 3 d. After evaporation of the solvent, the residue was dissolved in methanol (20 mL) and poured into diethyl ether (200 mL). The product was collected by centrifugation to give $PyC_{10}Py$ in 91% yield as a brown solid.

^{1}H NMR (D_2O, 500 MHz): δ 8.90 (d, J = 6.6 Hz, 4H, 2-position of pyridine), 8.62 (t, J = 8.2 Hz, 2H, 4-position of pyridine), 8.14 (t, J = 7.7 Hz, 4H, 3-position of pyridine), 4.67 (t, J = 7.3 Hz, 4H, α methylene in decamethylene), 2.08 (m, 4H, β methylene in decamethylene), 1.42–1.30 (m, 12H, χ, δ, ε methylene in decamethylene).

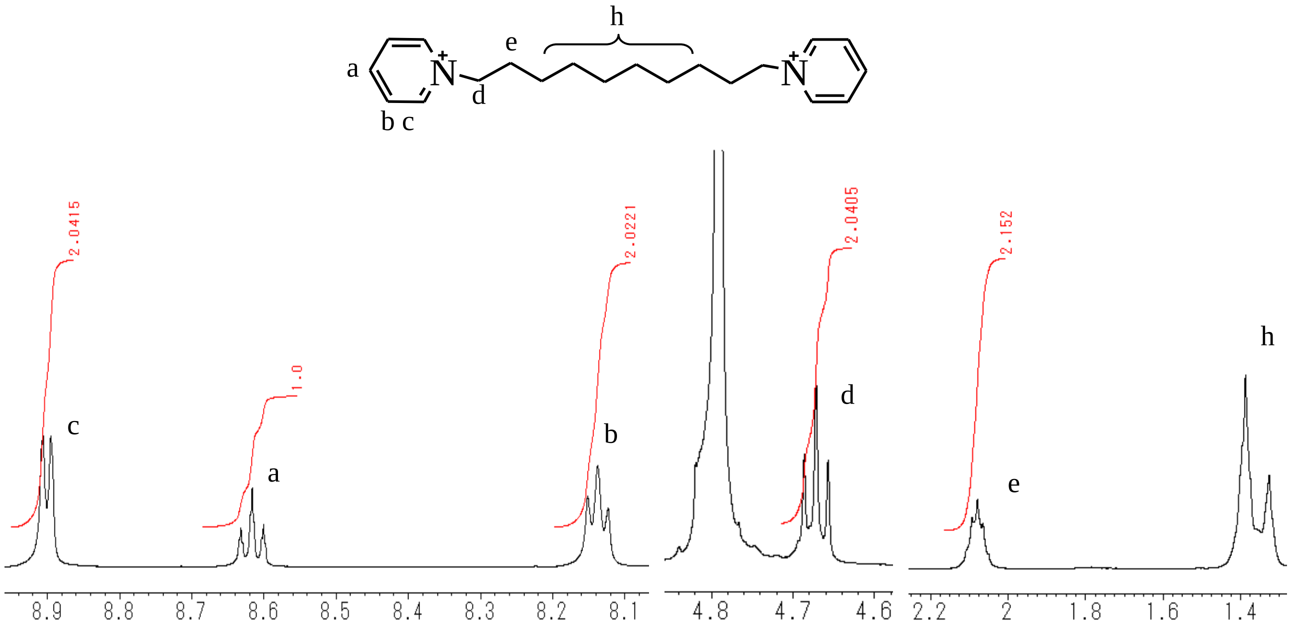

Figure S2: 500 MHz ^{1}H NMR spectra of $PyC_{10}Py$ in D_2O at 30 °C.

Preparation of α,α-CD dimer

6-NH_2- α-CD (120 mg, 0.12 mmol) and terephthalic acid (8.0 mg, 0.50 mmol) were dissolved in dried DMF (20 mL). DMT-MM (34 mg, 0.12 mmol) was added and the mixture was stirred at rt for 4 d. After evaporating the solvent, the residue was dissolved in water (10 mL) and poured into acetone (100 mL). The product was collected and purified by reversed-phase chromatography (elution: water-acetonitrile) to give α,α-CD dimer as a white solid in 22% yield.

^{1}H NMR (DMSO-d_6, 500 MHz): δ 8.36 (t, 2H, -N*H*), 7.88 (s, 4H, *Ph*), 5.59-5.40 (m, 24H, O(2, 3)*H* of α-CD), 4.97-4.78 (m, 12H, C(1)*H* of α-CD), 4.55-4.41 (m, 10H, O(6)*H* of α-CD), 3.84–3.48 (m, C(6,3,5,2,4)*H* of α-CD); MALDI–TOF *m/z*: 2095 [M + Na]$^+$.

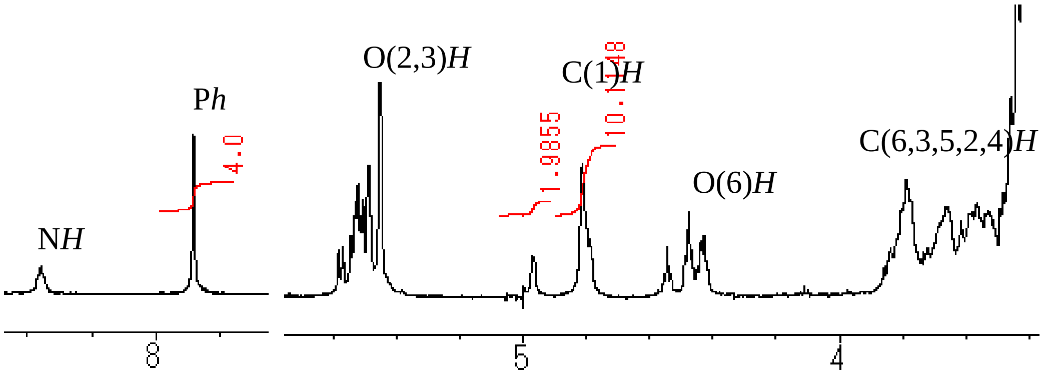

Figure S3: 500 MHz ^{1}H NMR spectra of α,α-CD dimer in DMSO-d_6 at 30 °C.

Preparation of α,β-CD dimer

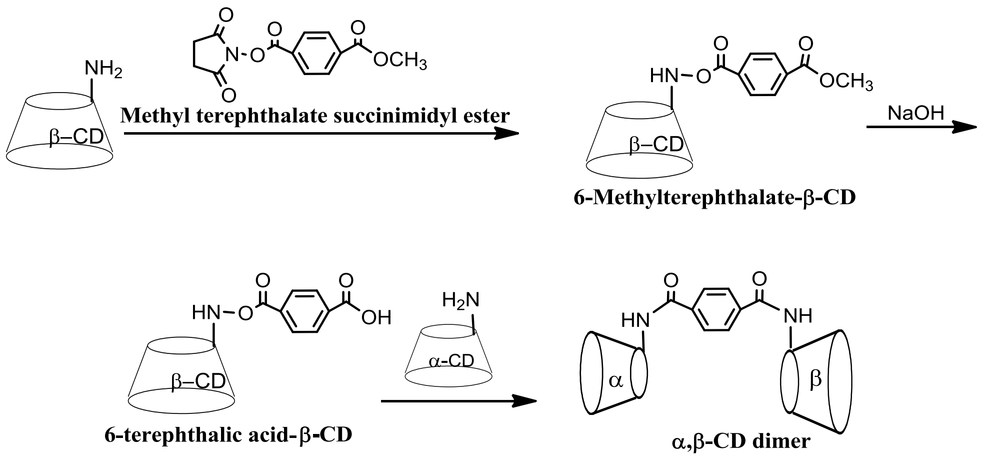

a) Terephthalate-β-CD

To a solution of 6-NH_2-β-CD (566 mg, 0.50 mmol) in dried DMF (7.0 mL) was added terephthalic acid methyl ONSu ester (138 mg, 0.50 mmol). After being stirred for 2 d at rt, the solution was poured into acetone (100 mL) to give terephthalate- β-CD as a yellow solid in 43% yield.

^{1}H NMR (DMSO-d_6, 500 MHz): δ 8.46 (t, 1H, -N*H*), 8.00 (d, 2H, *Ph*), 7.95 (s, 3H, -CH_3), 7.94 (d, 2H, *Ph*), 5.83–5.59 (m, 14H, O(2, 3)*H* of β-CD), 4.95–4.79 (m, 7H, C(1)*H* of β-CD), 4.45–4.32 (m, 6H, O(6)*H* of α-CD), 3.74–3.51 (m, C(3,6,5,3,4)*H* of α-CD); TLC: R_f 0.22 (*n*-butanol/ethanol/water 5:4:3).

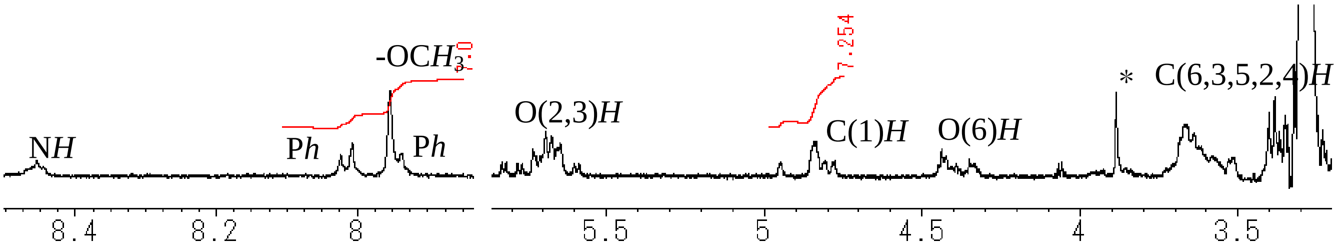

Figure S4: 500 MHz ^{1}H NMR spectra of 6-terephthalate-β-CD in DMSO-d_6 at 30 °C.

b) Terephthalic acid-β-CD

To a solution of 6-terephthalate ester-β-CD (605 mg, 0.47 mmol) in water (120 mL) was added NaOH (0.1 M, 7.0 mL). After being stirred for 12 h at rt, the solution was concentrated and purified by DIAION HP-20 column. The column was flushed with water (500 mL) and then eluted with water/methanol 80:20 (v/v). The fraction was concentrated to give 6-terephthalic acid-β-CD as a yellow solid in 70% yield.

^{1}H NMR (DMSO-d_6, 500 MHz): δ 8.39 (t, 1H, -NH), 7.98 (d, 2H, Ph), 7.90 (d, 2H, Ph), 5.83–5.59 (m, 14H, O(2, 3)*H* of β-CD), 4.95–4.79 (m, 7H, C(1)*H* of β-CD), 4.45–4.32 (m, 6H, O(6)*H* of β-CD), 3.74–3.51 (m, C(3,6,5,3,4)*H* of β-CD); TLC: R_f 0.32 (*n*-butanol/ethanol/water 5:4:3).

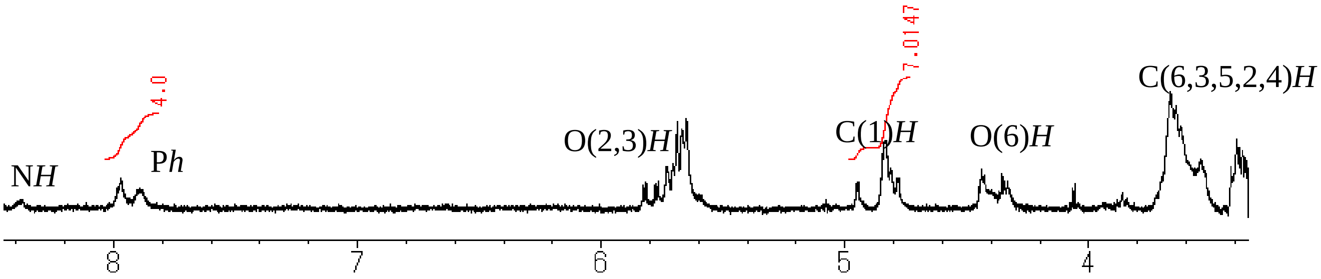

Figure S5: 500 MHz ^{1}H NMR spectra of 6-terephthalic acid-β-CD in DMSO-d_6 at 30 °C.

c) α,β-CD dimer

The synthetic procedure was the same as for the α,α-CD dimer, using 6-terephthalic acid-β-CD (65 mg, 0.050 mmol), 6-NH_2-α-CD (59 mg, 0.060 mmol), DMT-MM (17 mg, 0.060 mmol), dried DMF (8 mL) to give α,β-dimer in 36% yield as a white solid.

^{1}H NMR (DMSO-d_6, 500 MHz): δ 8.32, 8.27 (m, 2H, -NH), 7.89 (s, 4H, Ph), 5.80–5.44 (m, 26H, O(2, 3)*H* of CD), 4.97–4.78 (m, 13H, C(1)*H* of CD), 4.53–4.35 (m, 11H, O(6)*H* of CD), 3.86–3.37 (m, C(3,6,5,3,4)*H* of α-CD); TLC: R_f 0.04 (*n*-butanol/ethanol/water 5:4:3); MALDI–TOF *m/z*: 2259 $[M + Na]^+$.

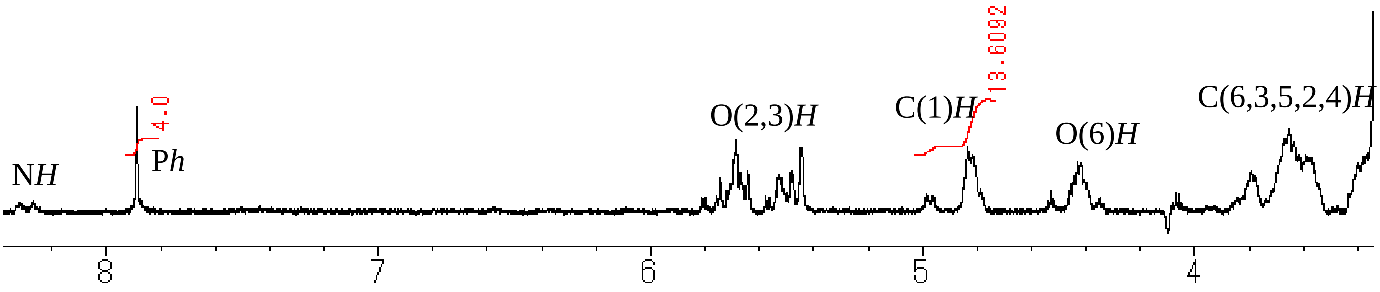

Figure S6: 500 MHz ^{1}H NMR spectra of α,β-CD dimer in DMSO-d_6 at 30 °C.

^{1}H NMR spectra of VP in the presence of α-CD or β-CD

Figure S7 shows the ^{1}H NMR spectra of VP in the presence of α-CD or β-CD. The decamethylene and pyridyl protons in VP/α-CD exhibit peak splitting, whereas those in VP/β-CD display peak shifts and broadening, indicating that the association–dissociation equilibrium between VP and β-CD is fast compared to the NMR time scale. The fast dissociation rate leads to a short lifetime of the VP/β-CD

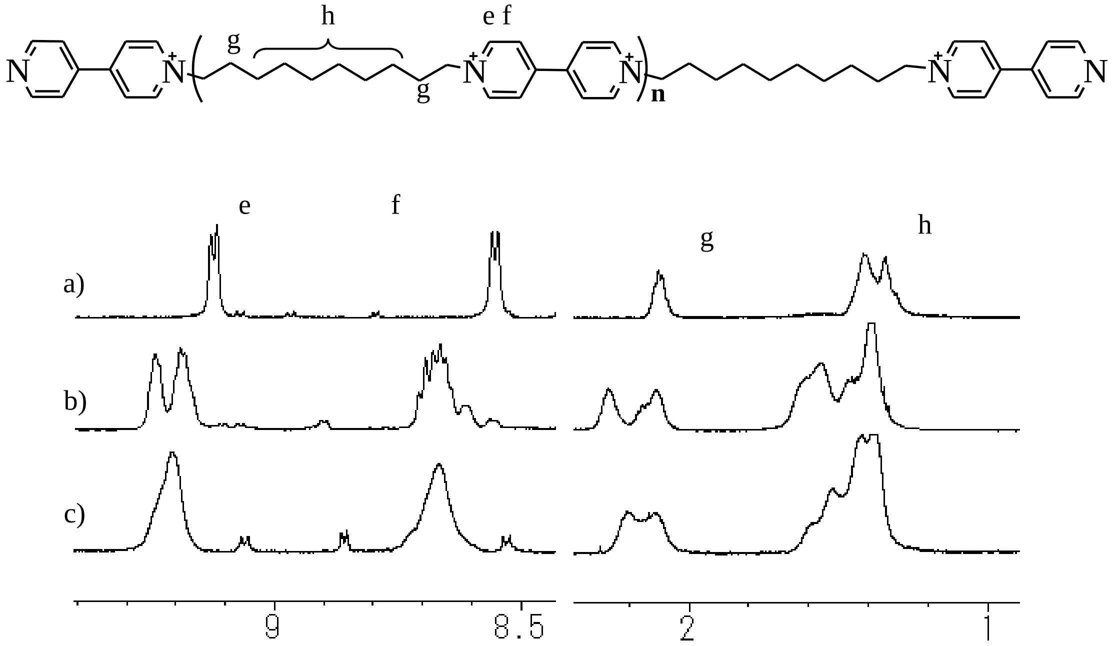

Figure S7: 500 MHz ^{1}H NMR spectra of VP (VP unit = 2mM) a) in the absence of CD; b) in the presence of α-CD (VP unit : CD = 1:2); c) β-CD(VP unit : CD = 1:2) at 30 °C in D_2O.
